# Metacogntive training add-on to esketamine treatment in TRD patients: effects on depressive rumination

**DOI:** 10.1192/j.eurpsy.2025.1360

**Published:** 2025-08-26

**Authors:** F. Raffone, D. Atripaldi, S. Testa, R. Cerlino, V. Martiadis

**Affiliations:** 1 Department of Mental Health, Asl Napoli 1 Centro; 2 Department of Advanced Medical and Surgical Sciences, Università degli Studi della Campania “L. Vanvitelli”, Naples, Italy

## Abstract

**Introduction:**

Depressive rumination has been the subject of increasing clinical and research interest in recent years. Numerous studies have demonstrated its central role in the etiopathogenesis and maintenance of depressive disorders. It refers to the struggle to control repetitive and passive thoughts with a hyper-focus on depressive symptoms, their causes, meanings and consequences. It is, therefore, a process that is often active in people with depressive mood and that can exacerbate and prolong depressive symptoms by promoting their chronicity. It can therefore be argued that depressive rumination may contribute to treatment resistance. The metacognitive model of major depressive disorder and its derived treatment focuses on rumination supporting beliefs and on their modification, with the aim of reducing their negative effects.

**Objectives:**

The aim of this study was to evaluate the improvement in depressive rumination using the metacognitive approach in TRD patients treated with intranasal esketamine.

**Methods:**

Twenty-five patients (13F) with a mean age of 55.88 years (±11.31) diagnosed with treatment-resistant major depression (TRD) received an 8-session weekly metacognitive training (MCT) intervention in addition to the standard intranasal esketamine treatment protocol. Patients were assessed at baseline and after 3 and 6 months of treatment using the Penn State Worry Questionnaire (PENN) and the Ruminative Response Scale (RRS) to assess depressive rumination, and the Montgomery-Asberg Depression Rating Scale (MDRS) to assess depressive symptomatology.

**Results:**

At baseline MADRS and RRS total scores did not differ significantly by gender or age. To assess the effect of MCT as an adjunct to esketamine therapy, a repeated measures ANOVA was performed comparing participants at different time points (T0, T3, T6). The analysis showed an effect of treatment on MADRS total score (η²=0.45; F=18.22, p=0.001), MADRS item 9 (pessimistic thoughts) (η²=0.18; F=4.85, p=0.02) and RRS total score. Post-hoc comparisons were significant for MADRS and RRS total scores, with progressive decreases between T0 vs T3, T0 vs T6 and T3 vs T6; for item 9, comparisons were significant between T0 vs T3 and T0 vs T6, with stability between T3 and T6. MCT treatment combined with intranasal esketamine resulted in significant improvements in depressive symptoms. It also reduced depressive rumination and maladaptive metacognitive beliefs.

**Conclusions:**

These preliminary results show that MCT as an adjunct to esketamine treatment is effective for depressive rumination, with stable results up to 6 months after treatment. The generalisability of the results is limited by the lack of a control group and the relatively small sample size. Further studies in larger populations and comparing MCT with other psychotherapies or usual care are needed to confirm these findings.

**Disclosure of Interest:**

None Declared

